# Esophageal Perforation During Transesophageal Echocardiography Managed Conservatively: A Case Report With a Review of the Literature on Management

**DOI:** 10.7759/cureus.75048

**Published:** 2024-12-03

**Authors:** Sathyatej Kosuri, Perumalla Hima Sanjana, Talal Asif

**Affiliations:** 1 Biomedical Sciences, University of Missouri Kansas City School of Medicine, Kansas City, USA; 2 Internal Medicine, University of Missouri Kansas City School of Medicine, Kansas City, USA; 3 Cardiology, University of Missouri Kansas City School of Medicine, Kansas City, USA

**Keywords:** conservative medical management, esophagram, pneumomediastinum, transoesophageal echo, traumatic esophageal perforation

## Abstract

Transesophageal echocardiography (TEE) is one of the cornerstones of cardiac imaging in inpatient and intra-operative settings. TEE is considered a safe procedure, but it may result in serious complications, such as esophageal injury, vocal cord paralysis, arrhythmia, hypotension, seizure, and cardiac arrest. Herein, we discuss one of the rare complications, esophageal perforation, and a conservative approach to managing the patient in a 64-year-old female who underwent a TEE prior to a scheduled valvular surgery. The same day, she returned to the emergency department with complaints of neck and left-sided chest pain. Further evaluation revealed an esophageal perforation. It was managed conservatively, with the use of antibiotics and nil-by-mouth placement. This case demonstrates that depending on the size of the defect, conservative approaches of management are a reasonable option and that not all cases will necessitate an emergent surgery.

## Introduction

Transesophageal echocardiography (TEE) is an essential imaging modality that plays a vital role in diagnosing and treating many cardiac conditions. Even though it is widely used and has a low-risk profile, it is still an invasive procedure with innate risks of complications, which include but are not limited to esophageal injury, vocal cord paralysis, arrhythmia, hypotension, seizure, and cardiac arrest. Esophageal perforation is a notably feared complication and is generally perceived to be a surgical complication by cardiologists in community [1]. However, in this case report, we present a patient who was managed with a conservative approach and recovered fully. We aim to review and provide algorithmic diagnostic and management approaches to guide physicians when encountering such patients.

## Case presentation

**Figure 1:**
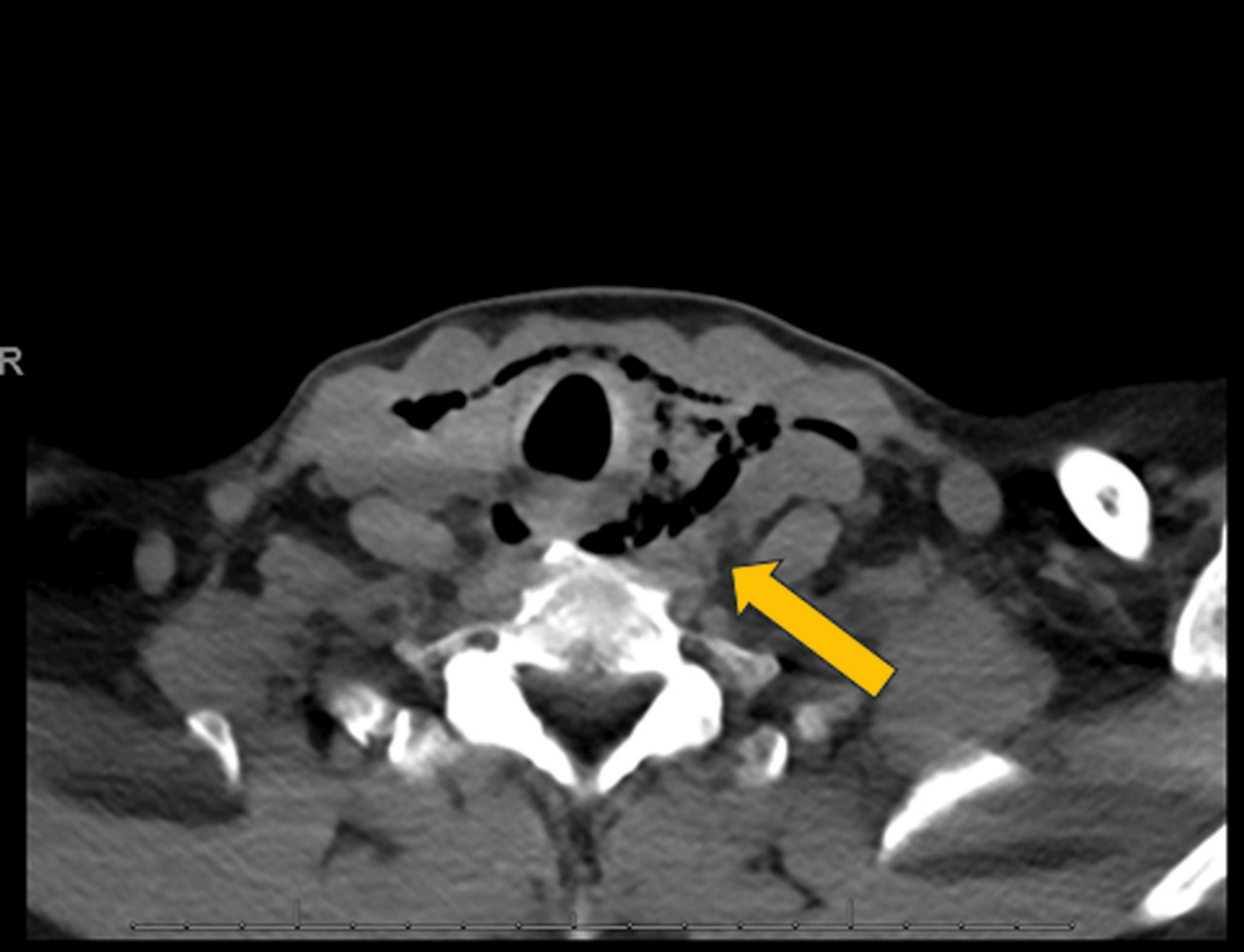
Computed tomography (CT) neck with oral contrast Extensive soft tissue air within neck with mild pneumomediastinum concerning for esophageal perforation.

**Figure 2:**
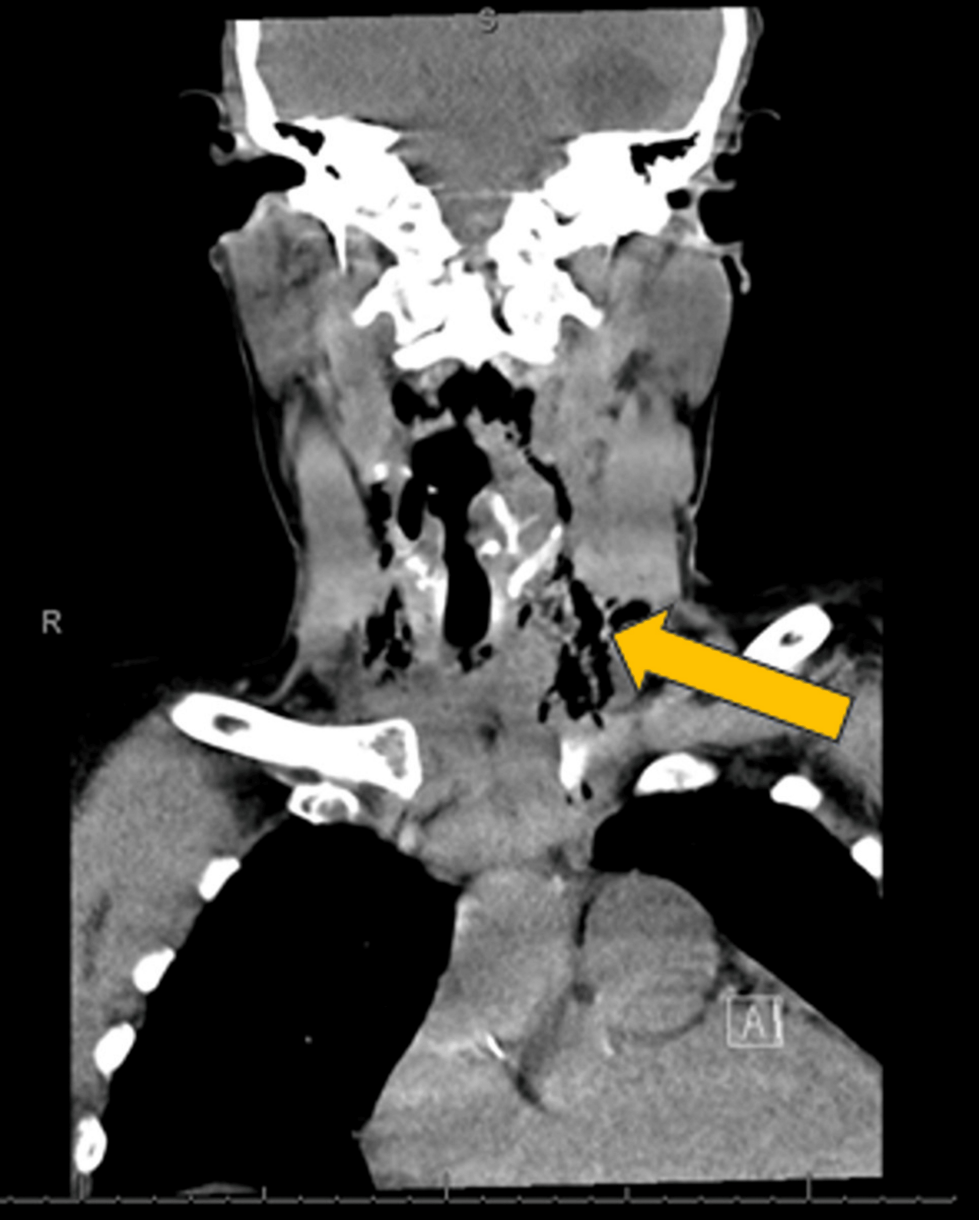
Computed tomography (CT) neck soft tissue Soft tissue air within soft tissue of neck

A 64-year-old female with a past medical history significant for severe mitral regurgitation, moderate aortic regurgitation, heart failure with a mildly reduced ejection fraction of 40 to 45%, hypertension, hyperlipidemia, presented to the emergency department (ED) with shortness of breath, neck pain and left-sided chest pain. She was unable to tolerate diet. Patient started having these symptoms two to three hours after undergoing a pre-operative TEE earlier in the day as part of a pre-operative evaluation for mitral valve repair and aortic valve replacement surgery. Her symptoms progressively worsened which made her present to the hospital. In the ED, she was in sinus tachycardia with a heart rate of 107 beats per minute, blood pressure of 139/60 mmHg, and was afebrile with an oxygen saturation of 95% on room air. Computed tomography (CT) of the neck with oral contrast revealed a perforation of the upper thoracic esophagus with two discrete perforations of 1.2 cm and 1 cm. In addition, trace pneumomediastinum and supraclavicular deep soft tissue air were seen (Figure 1, Figure 2). She was started on vancomycin and piperacillin-tazobactam empirically. The patient was placed nil-by-mouth, and oral medications were held.

**Figure 3:**
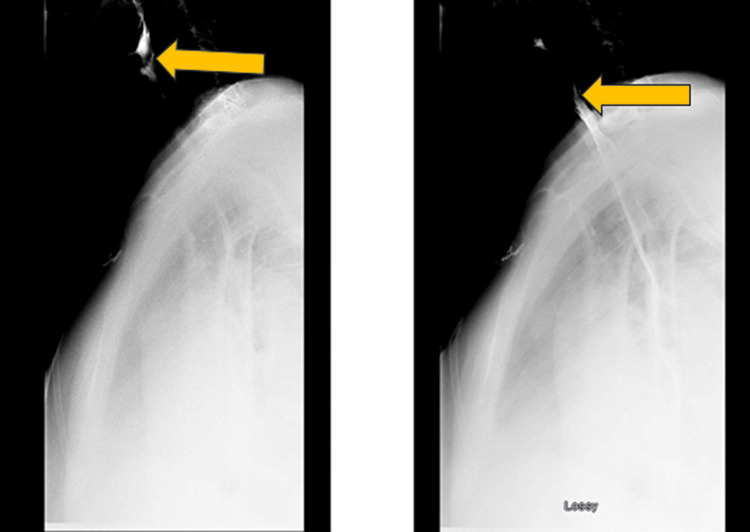
Esophagram No residual perforation observed on esophagram.

**Figure 4:**
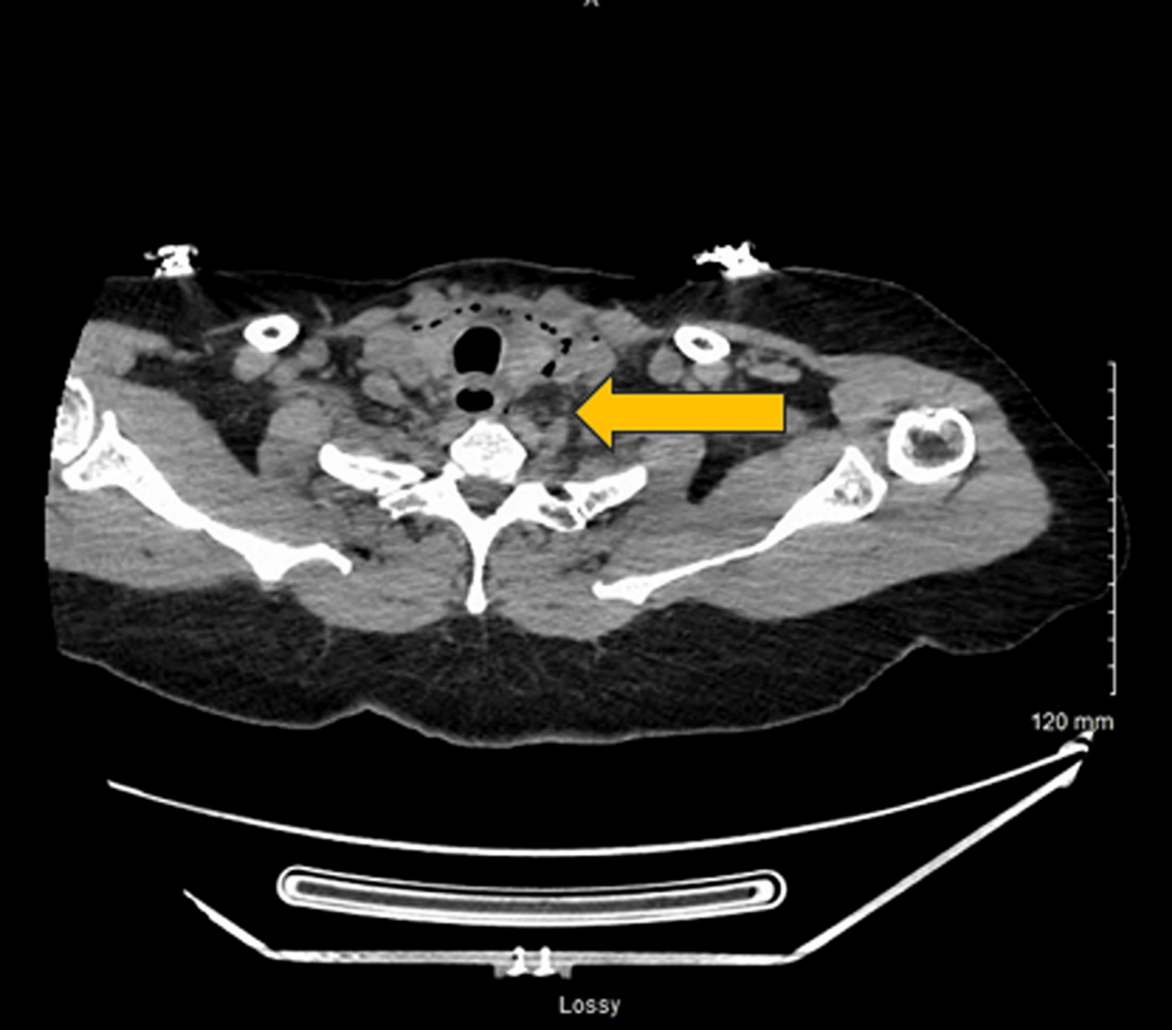
Repeat Computed tomography (CT) Neck Improvement in the air in the soft tissues of the neck.

The patient then underwent an esophagram. The esophagram did not show any signs of persistent perforation (Figure 3). A multidisciplinary team consisting of gastroenterology, ENT, and cardiothoracic surgery decided to manage the patient conservatively. A clear liquid diet was initiated, which was well tolerated by the patient. A follow-up CT of the neck and chest with oral contrast showed no evidence of residual esophageal perforation (Figure 4). The diet was advanced to soft foods, and her home medications were re-initiated. At this time, the multidisciplinary care team decided that empiric antibiotics were no longer necessary in managing this patient, and vancomycin and piperacillin-tazobactam were discontinued. She was hemodynamically stable, tolerating oral intake, and ambulating appropriately, so she was deemed eligible for discharge. The patient underwent mitral valve repair and aortic valve repair a week later with no complications. No intraoperative TEE was performed during surgery. The patient is doing well on follow-up.

## Discussion

TEE is a commonly used cardiac imaging modality. It is a fairly safe and well-tolerated semi-invasive procedure with a 3% complication rate and a reported 0.2% mortality rate [2-4]. Risks that are commonly associated with TEE are dysphagia, hoarseness, lip injury and dental injury [4]. However, it may result in serious complications, such as esophageal injury, vocal cord paralysis, arrhythmia, hypotension, seizure, and cardiac arrest. An upper gastrointestinal tract injury is a rare, devastating complication of TEE. The incidence of iatrogenic esophageal perforation by TEE is 0.03-0.09%. The mortality of gastrointestinal perforation ranges from 10% to 56% [5,6]. Delayed detection of esophageal perforation may lead to mediastinitis, severe sepsis, and death. To avoid these complications, the American Society of Echocardiography (ASE) provides a comprehensive list of absolute and relative contraindications, review of which is essential as part of pre-procedural patient evaluation [4].

In general, esophageal perforations often have vague presentations with symptoms such as neck pain, dysphagia, odynophagia and possible fevers and chills [7]. On physical exam, crepitus may be present at or near the neck on palpation. These symptoms, with a history of a recent endoscopic procedure, indicate a need for urgent testing to rule out esophageal perforation. As a physician practicing in the community, it is important to know that radiographic imaging modalities formulate the cornerstone of diagnostic imaging. No single imaging modality is the gold standard, and perforation can be missed, given the limited sensitivity of each modality. Additional imaging should be guided by the index of suspicion. CT with oral contrast is safe as iodine is water soluble, is readily available and should be pursued initially [4]. For anatomical localization, CT neck and chest is used [4]. A plain X-ray can detect subcutaneous emphysema along with air in the mediastinum which would indicate a perforation in the thoracic esophagus [8]. Contrast esophagography is often used as a confirmation tool, as seeing leakage of contrast out of the esophageal space into the mediastinal space would indicate a perforation [3]. A caveat here is that water studies are often used instead of barium studies, as barium carries an increased risk of chemical mediastinitis [3]. Flexible esophagoscopy in the hands of an experienced endoscopist is considered safe [4]. We were able to localize the perforation using the CT scan, and we used esophagram as a tool to confirm healing. 

Initial management includes nil by mouth and broad-spectrum antibiotics. Nasogastric tube should not be inserted blindly. For community practitioners, it is imperative to involve surgical specialties as a multidisciplinary approach is safest due to the complexity of the injury. Management depends on the extent of the trauma. Surgical management of esophageal perforations has been conceived by physicians as the mainstay of therapy for many years. If a patient has extensive trauma and large perforation (defined as > 2 cm by American Association for the Surgery of Trauma), surgical debridement is often considered followed by primary repair of the lesion [4,6]. The size of the perforation in our patient is less than 2 cm, therefore we proceeded with conservative management. Endoscopically placed stents and clips are an additional therapeutic option in experienced centers; however, data is limited on the efficacy of this treatment compared to surgical treatment [9]. We aim to bring to the attention of community physicians and cardiologists, that conservative, non-operative management is a practical management approach in appropriately selected cases. This is a worthwhile approach in patients who have small iatrogenic perforations that are contained and who do not demonstrate signs of sepsis [10]. It is important to remember that these injuries are non-traumatic iatrogenic injuries and do not disrupt tissue planes [11]. Therefore, the chance of spontaneous seal, whether in the cervical or thoracic esophagus, is higher, with a higher probability of absence of spillage. Conservative management allows the esophagus to heal on its own while preventing any infections from occurring with antibiotics and preventing possible further damage to the esophagus and surrounding tissue by stopping oral intake. A follow-up esophagram before the resumption of feeding is required. There is no data on how long to wait. However, this decision is guided by the clinical status of the patient.

## Conclusions

With our case, we add to the literature the feasibility of conservative management in this feared complication of TEE-induced esophageal perforation. This can be a practical management approach in carefully selected cases with small perforations and low infection concern to avoid surgical risks. Even though community cardiologists perform TEEs on a regular basis, this complication is so rare that most physicians will not see a case in their entire career which makes management decisions challenging. Therefore, this case also aims to provide guidance to physicians practicing especially in community settings.
